# Perceptions of Saudi parents of students with autism toward the responsibilities of transition plan members in implementing transition plans

**DOI:** 10.1371/journal.pone.0345501

**Published:** 2026-03-20

**Authors:** Salman Almughyiri

**Affiliations:** Department of Special Education, College of Education in Al-Kharj, Prince Sattam bin Abdulaziz University, Al-Kharj, Saudi Arabia; Hacettepe University: Hacettepe Universitesi, TÜRKIYE

## Abstract

**Objective:**

This study investigates how Saudi parents of children with autism perceive the roles and responsibilities of school staff and families in implementing transition plans. It looks at how these duties are shared between home and school during the planning phase.

**Methods:**

A cross-sectional, quantitative approach was employed. A total of 469 parents of students with autism from the Riyadh (n = 243) and Makkah (n = 226) regions completed a structured survey. The instrument, developed from validated measures and based on the National Technical Assistance Center on Transition (NTACT) practices, used a 5-point Likert scale. Descriptive and inferential statistics (t-tests and cross-tabulations) were applied to compare perceptions across demographic variables such as gender, education level, and region.

**Results:**

Parents viewed schools as more responsible than homes for most transitional skills, especially in areas like self-advocacy, vocational preparation, and study skills. Conversely, daily living and independent living skills were more often considered the family’s responsibility. Perceptions did not significantly differ based on gender, education, or location.

**Conclusion:**

The results indicate a collaborative responsibility between families and schools, with schools being considered the primary partner in most skill areas. To support smooth transitions for individuals with autism, increased collaboration between educators and families and parent-led training are essential.

## Introduction

Transition planning is widely recognized as a critical component of special education services for students with autism spectrum disorder (ASD), particularly in supporting successful movement from school to post-school life. Internationally, transition plans are designed to address postsecondary education, employment, independent living, and community participation through coordinated efforts among schools, families, and related service providers [1]. These plans are typically implemented through collaborative approaches that emphasize shared responsibility between educational systems and families, with parents playing an essential role in supporting skill development beyond the school environment. Research conducted in international contexts [2] highlights the importance of parental involvement in both planning and implementing transition services for students with autism. When parents are actively engaged and supported, transition plans are more likely to align with students’ interests, abilities, and long-term goals. However, the literature also indicates that parental responsibilities during transition implementation are not always clearly defined and may vary depending on policy frameworks, service structures, and cultural contexts, which can create uncertainty regarding the distribution of responsibilities between schools and families [3].

From a conceptual perspective, parental involvement in transition planning and implementation can be understood through ecological and person-centered approaches. Ecological Systems Theory emphasizes that students’ development is influenced by interactions across multiple systems, including family, school, and community, positioning parents as key contributors within the child’s immediate and interconnected environments [4]. In addition, person-centered transition frameworks highlight the importance of aligning transition goals and implementation with students’ individual strengths, preferences, and family contexts. Within this perspective, parents play a central role in supporting transition-related skills beyond the school setting and in reinforcing continuity between home and school. Together, these perspectives provide a conceptual basis for examining parental perceptions of responsibility in implementing transition plans [5].

Within the Saudi Arabian context, special education services have undergone significant advancements over the past two decades, primarily due to improvements in accessibility and capacity. According to [1], care for students with special needs has expanded to include programs addressing autism, intellectual disabilities, visual impairments, and hearing impairments. In addition, studies have documented notable growth in special education departments at tertiary institutions in Saudi Arabia, contributing to an increase in the number of trained special education teachers and academic programs [2,3]. A guide released by the Saudi Health Council’s National Center for Developmental Behavioral Disorders (NCCDD) identifies autism spectrum disorder as a significant health and educational concern in Saudi Arabia [6]. Despite these developments, research addressing autism remains limited, particularly with regard to policies and practices related to transition planning and implementation, leading many local practices to rely on evidence generated in developed countries. Although research on students with autism in Saudi Arabia has increased since approximately 2008, notable gaps remain, especially in relation to transition services and the implementation of transition programs. Existing literature points to inconsistencies between policy intentions and actual practices in transition planning [7]. Furthermore, transition services are primarily concentrated in major urban areas, which limits access for families in other regions of the country [6]. Previous studies have also reported that available transition services do not consistently meet the needs of families of students with autism, and that transition plans are often perceived as difficult to implement in practice [7]. These concerns highlight the need for further investigation into how transition planning and implementation are understood and experienced by families, in order to inform the development of more practical and responsive transition services [8]. The transition plan includes post-high-school goals that are designed and determined based on students’ interests, talents, needs, and abilities [9]. Planning and implementing transition programs are essential for supporting students with autism as they prepare for adult life. Transition services contribute to reducing barriers to employment, postsecondary education, and vocational training, thereby supporting broader social and economic participation [10]. Despite progress in expanding transition-related services, implementation remains uneven, and many students with autism continue to experience difficulties during the transition from school to adult life. This situation reflects an ongoing gap between policy and practice in transition planning and implementation. Similar challenges have also been observed in transition planning for individuals with other disabilities, particularly at the caregiver level [11]. Parental involvement is especially important in the implementation of transition plans, as many transition-related skills are developed and reinforced outside the school environment. Parents often begin supporting transition-related skills early in their child’s educational journey, and transitions across educational stages represent critical periods for establishing effective service pathways [12–16]. However, despite parental expectations regarding transition outcomes, the process can be stressful and demanding due to limited communication with schools, insufficient follow-up, and a lack of structured support during implementation. Available literature suggests that transition services for students with autism in Saudi Arabia are not consistently implemented across regions. Transition programs are primarily concentrated in major cities, while families in other areas may experience limited access to services. In addition, gaps between policy intentions and actual practices have been noted, particularly in relation to the implementation of transition plans. Despite national commitments to inclusive education and disability support articulated in Saudi Vision 2030 and the Ministry of Education’s Special Education Strategy (2022), transition services for students with autism remain unevenly implemented across regions, particularly at the level of family involvement and plan execution. Parents have reported concerns regarding the practicality of transition plans and the extent to which schools provide guidance and follow-up during the transition process.

Previous research has shown that many parents of students with autism do not actively participate in transition processes due to challenges such as limited time, insufficient knowledge of transition services, and unclear role expectations [17]. In addition, schools may not consistently seek proactive parental involvement or provide adequate guidance regarding transition responsibilities. Studies have also indicated that person-centered transition planning and implementation, particularly when carried out at the caregiver level, remain inconsistent and require further improvement [18,19]. These findings reinforce the view that parents play a vital role in transition planning and implementation for students with autism [20]. Despite the recognized importance of collaboration between families and schools, parents continue to face challenges that limit their involvement in planning and executing transition programs. These challenges highlight the need for greater guidance and support for parents, as well as clearer communication regarding responsibilities among transition plan members. At the same time, there remains a lack of empirical evidence examining parents’ perceptions of the responsibilities associated with implementing transition plans for students with autism in Saudi Arabia, particularly in relation to the gap between policy and practice [21].

Accordingly, the present study aims to explore Saudi parents’ perceptions of the responsibilities of transition plan members in implementing transition plans for students with autism. By examining how parents perceive the distribution of responsibilities between schools and families, this study seeks to contribute evidence that may inform efforts to strengthen collaboration and improve transition practices within the Saudi educational context.

### Research aim and rationale

The main goal of this study is to examine how parents of students with autism view their responsibilities in enforcing transition plans in Saudi Arabia. According to background information, parents play an essential role in planning and implementing transition plans [22]. However, their involvement and contribution are limited due to challenges such as a lack of support, knowledge, and guidance regarding transition services [23]. This study seeks to explore parental perceptions in this area to help develop practical policies that support the effective implementation of transition services.

### Research questions

1 What is the perceived level of responsibility among parents of students with autism in Saudi Arabia for implementing transition plans both inside and outside of schools?
2 What are the differences between school and home responsibilities during transition?
3 Are there any statistically significant differences in parents’ perceptions of their level of implementation of transition plans for students with autism based on parents’ gender, region, or educational level?

## Methodology

### Materials

A structured survey instrument was used to assess parental perceptions of responsibility for implementing transition planning services at both school and family levels [24]. The instrument was adapted from previously validated transition-related measures [25,26] and aligned with evidence-based practices recommended by the National Technical Assistance Center on Transition (NSTTAC) [9,27]. Item selection was informed by a review of experimental and applied research on secondary transition skills [28].

All original items were retained except for the “one more than” instructional strategy, which was excluded due to limited applicability within the Saudi educational context. In addition, two items were adapted from [29] to enhance contextual relevance. Specifically, references to self-determined learning instructional strategies were revised to reflect self-determination skills more broadly, consistent with local educational practices for students with autism.

The instrument underwent a structured translation process to ensure linguistic and conceptual equivalence. Items were translated into Arabic and reviewed by bilingual experts in special education, followed by reconciliation of discrepancies to improve clarity and cultural appropriateness [30]. A pilot pre-test was conducted to evaluate item clarity and relevance in relation to the study objectives.

The final survey consisted of two sections. The first section collected demographic information, including parent gender, educational level, and region of residence. The second section included 26 items (see supplementary S1 Table) measuring perception levels of responsibility for implementing transition planning practices. Responses were rated on a five-point Likert scale ranging from 1 (very low) to 5 (very high). Table 1 presents the interpretation of Likert scale levels.

**Table 1:** Interpretation of Likert Scale Levels.

Likert Range	Interpretation
1.00–1.79	Very Low Responsibility
1.80–2.59	Low Responsibility
2.60–3.39	Moderate Responsibility
3.40–4.19	High Responsibility
4.20–5.00	Very High Responsibility

Ethical approval for the study was obtained from the Standing Committee of Bioethics Research at Prince Sattam bin Abdulaziz University (SCBR-89/2024) on July 19, 2024, following authorization from relevant educational authorities.

### Validity

Content and construct validity were addressed through multiple procedures. During the pilot phase, the instrument was reviewed by 11 faculty members specializing in special education to assess item relevance, clarity, and alignment with transition planning constructs. Their feedback resulted in minor wording refinements to enhance content validity.

Internal construct consistency was examined statistically by analyzing inter-item correlations across the scale. Correlation coefficients supported acceptable internal structure, as presented in Table 2.

**Table 2:** Correlation coefficients between survey Items and the survey domain.

*Item*	*Correlation Coefficient*	*Item*	*Correlation Coefficient*	*Item*	*Correlation Coefficient*
1	0.706	10	0.745	19	0.687
2	0.713	11	0.716	20	0.710
3	0.757	12	0.653	21	0.669
4	0.742	13	0.639	22	0.688
5	0.774	14	0.677	23	0.671
6	0.675	15	0.755	24	0.736
7	0.743	16	0.764	25	0.724
8	0.687	17	0.786	26	0.751
9	0.657	18	0.715		

** for a significance level of 0.01

While confirmatory factor analysis was not conducted due to the applied nature of the study and its focus on group-level comparisons, the instrument demonstrated coherence consistent with its theoretical dimensions of school and family responsibility.

Data management procedures included screening for incomplete responses, duplicate entries, and missing values to ensure data accuracy and integrity [31].

### Reliability

Internal consistency reliability was assessed using Cronbach’s alpha. Reliability coefficients were calculated separately for each dimension of the instrument. The school responsibility subscale demonstrated acceptable internal consistency (α = [0.93]), and the family responsibility subscale also met recommended reliability thresholds (α = [0.91]). These values exceed the commonly accepted criterion of α ≥ .70 for research instruments [32]. Reliability results are presented in Table 3.

**Table 3:** Cronbach’s Alpha for Instrument Reliability.

Scale/ Domain	Cronbach’s α
Full Scale	0.95
School Responsibility Subscale	0.93
Family Responsibility Subscale	0.91

**Note:** All values exceed the 0.90 benchmark for high internal reliability.

### Participants

Participants were parents of students diagnosed with autism spectrum disorder and enrolled in public schools in the Riyadh and Makkah regions of Saudi Arabia. Inclusion criteria required that parents have a child formally identified with autism by school or medical records and currently receiving special education services. Parents of students with other disability categories were excluded to maintain population specificity.

A total of 469 parents participated in the study. Participant lists were obtained from Departments of Special Needs within each region and served as the sampling frame. Within these lists, parents were selected using a random selection procedure proportional to the size of each regional population. Although the sampling relied on existing school records, random selection was applied within the available sampling frame, making the procedure best described as stratified random sampling by region [33].

### Recruitment

Following institutional approvals, school principals facilitated contact with eligible parents by sharing study information and invitation emails. Parents received a secure survey link via email and were provided with detailed information regarding the study’s purpose, procedures, confidentiality, and voluntary nature of participation [34]. Written informed consent was obtained electronically prior to survey access. Only parents participated in the study; no teachers or students were involved. Participants were informed of their right to withdraw at any time without penalty. Data collection occurred over approximately six weeks.

### Data collection and analysis

Data were analyzed using IBM SPSS Statistics (Version 26). Descriptive statistics, including means, standard deviations, and frequencies, were computed to summarize perception levels across school and family responsibility domains. Independent-samples t-tests were conducted to examine group differences based on parent gender, educational level, and region. Effect sizes (Cohen’s d) were calculated for all inferential comparisons. Statistical significance was evaluated using a two-tailed criterion of p < .05. Cases with more than 5% missing data were excluded using listwise deletion. The final dataset included 469 valid responses.

## Results

### Perception of the level of responsibility for implementation at the school and family/household level

Descriptive analysis was used to examine parental perceptions of the level of responsibility for implementing transition plans at both the school and family/household levels. Descriptive statistics, including means and standard deviations, were calculated to summarize responses and to rank perception levels based on the measurement scale, as explained in the instrumentation section. The items assessed responsibilities related to implementing transition plans at the school level and covered a range of skill areas, including study skills, problem-solving, vocational skills, goal setting, time management, self-advocacy, daily living skills, interpersonal skills, stress management, decision-making, social skills, independent living skills, and self-awareness. Overall, the perceived responsibility for implementing transition plans was higher at the school level (M = 3.68, SD = 1.01) compared to the family level (M = 3.18, SD = 0.96), indicating that parents generally viewed schools as having a greater role in transition plan implementation.

### Demographic characteristics

Frequency analysis was performed to describe the demographic characteristics of the participants, including gender, educational level, and geographic location. Regarding gender, more than half of the participants were female (52.24%, n = 245), while male parents accounted for 47.76% of the sample (n = 224). With respect to location, most participants were from the Riyadh region (51.81%, n = 243), followed closely by participants from the Makkah region (48.19%, n = 226). In terms of educational attainment, the majority of parents held an undergraduate degree (73.88%, n = 346, in comparison, 26.23% of the participants (n = 123) reported a high school education. These demographic characteristics are presented in Table 4.

**Table 4:** Demographic characteristics of the sample.

*Category*	*N*	*Percentage*
Gender		
Male	224	47.76
Female	245	52.24%
Education Level		
High School	123	26.23%
Undergraduate	346	73.88%
Region		
Riyadh	243	51.81%
Makkah	226	48.19%

### Ranking of transitional skills implementation based on parental perception

Using descriptive statistics, including means and standard deviations, parental perceptions of responsibility for implementing transitional skills within schools and at home were examined and ranked, as shown in Table 5. The results indicate that, overall, schools were perceived as having greater responsibility for teaching and implementing transitional skills than families.

**Table 5:** Ranking of Transitional skills by level of responsibility in implementation in school and outside school.

*Transition skills*	*Schools Responsibilities*		*Family Responsibilities*	
	*Mean*	*STD*	*Mean*	*STD*
**Self-advocacy skills**	3.94	1.13	2.98	1.04
**Study skills**	3.93	1.07	2.97	0.99
**Vocational skills**	3.88	0.87	2.94	0.82
**Daily living skills**	3.45	1.02	3.66	0.95
**Interpersonal skills**	3.82	0.91	3.44	1.02
**Stress-management skills**	3.60	1.04	3.22	1.05
**Problem-solving skills**	3.59	1.01	3.03	0.85
**Decision-making skills**	3.77	1.02	3.01	1.01
**Time management skills**	3.75	1.03	3.02	0.87
**Self-Awareness**	3.66	1.09	3.09	1.18
**Social skills**	3.60	0.85	3.46	1.02
**Independent living skills**	3.25	0.93	3.43	0.84
**Goal-setting and attainment skills**	3.65	1.03	3.19	1.12
	**3.68**	**1.01**	**3.18**	**0.96**

At the school level, self-advocacy was identified as the highest-rated responsibility (M = 3.94, SD = 1.13), followed by study skills (M = 3.93, SD = 1.07) and vocational skills (M = 3.88, SD = 0.87). At the family level, daily living skills were perceived as the most frequently implemented (M = 3.66, SD = 0.95), followed by interpersonal skills (M = 3.44, SD = 1.02). These findings suggest that parents view schools as primarily responsible for developing academic, self-advocacy, and career-related skills, whereas families are perceived to play a more substantial role in supporting daily living and interpersonal skills. A complete ranking of transitional skills across school and family contexts is presented in Table 5.

### Differences in perception level based on gender, educational level, and location

Inferential analyses were conducted to examine differences in parental perception levels regarding responsibility for implementing transition plans based on gender, educational level, and geographic location. Independent-samples t-tests were used to compare mean perception levels across groups.

With respect to gender, no statistically significant difference was found between male and female parents as shown in Table 6. Female parents reported slightly higher perception levels (M = 3.19, SD = 0.77) than male parents (M = 3.10, SD = 0.72); however, this difference was not statistically significant, *t*(467) = 0.70, *p* = .486, Cohen’s *d* = 0.12, indicating a small and practically negligible effect size. These results indicate that parental perceptions of responsibility for transition plan implementation were comparable across gender groups.

**Table 6:** The responses of male and female parents using the Independent Samples t-test.

Gender	Sample size	M	SD	T-value	Degree of freedom	p-value
Male	224	3.10	0.72	0.70	467	0.486
Female	245	3.19	0.77			

*Note.* Cohen’s *d* = 0.12, indicating a small effect size.

Similarly, an independent-samples t test was conducted to examine differences in perception levels based on parents’ educational attainment (high school vs. undergraduate) as shown in Table 7. The results showed no statistically significant difference between parents with a high school education (M = 2.91, SD = 0.88) and those with an undergraduate degree (M = 3.03, SD = 0.69), *t*(369) = 0.70, *p* = .457, Cohen’s *d* = 0.09, indicating a negligible effect size. These findings suggest that educational attainment did not meaningfully influence parents’ perceptions of responsibility for implementing transition plans.

**Table 7:** The t-test for independent samples was used to analyze parents’ answers about the variable of educational qualification.

Qualifications	Sample size	M	SD	T-value	Degree of freedom	p-value
High school	123	2.91	0.88	0.698	467	0.457
Undergraduate	346	3.03	0.69			

*Note.* Cohen’s *d* = 0.09, indicating a negligible effect size.

Finally, regional differences in parental perception levels were examined using an independent-samples *t*-test, as shown in Table 8. The results indicated no statistically significant difference between parents residing in Riyadh (M = 3.07, SD = 0.76) and those residing in Makkah (M = 3.11, SD = 0.81), *t*(467) = 0.79, *p* = .472, Cohen’s *d* = 0.05, indicating a trivial effect size. This finding suggests that parents’ perceptions of responsibility for transition plan implementation were similar across the two regions.

**Table 8:** The t-test for independent samples was used to analyze parents’ answers about the variable of region.

Experience	Sample size	M	SD	T-value	Degree of freedom	p-value
Riyadh	243	3.07	0.76	0.792	467	0.472
Makkah	226	3.11	0.81			

*Note.* Cohen’s *d* = 0.05, indicating a trivial effect size.

### Relationship between school and family responsibility

To further explore relational patterns between perceived responsibilities at the school and family levels, a Pearson correlation analysis was conducted between the overall school responsibility score and the overall family responsibility score. The analysis revealed a small but statistically significant positive association between perceived school responsibility and perceived family responsibility (r = .21, p < .001). This finding indicates that parents who perceive higher levels of responsibility at the school level also tend to perceive higher levels of responsibility at the family level, suggesting that school-based and family-based roles in transition plan implementation are viewed as complementary rather than competing.

## Discussion

The present study examined Saudi parents’ perceptions of responsibility for implementing transition plans for students with autism, with a particular focus on differences between school and family contexts. Overall, parents rated school responsibility significantly higher than family responsibility across most transition domains, especially in areas related to self-advocacy, academic skills, and vocational preparation. In contrast, family responsibility was perceived as more prominent in daily living and interpersonal skills. These findings indicate that parents view transition plan implementation as a shared process, with differentiated roles for schools and families rather than equal responsibility across settings.

The stronger emphasis placed on schools may be understood within broader cultural and systemic contexts. In Saudi Arabia, education systems are traditionally viewed as the primary authority responsible for structured instruction, professional guidance, and formal planning processes. Similar patterns have been reported in international research conducted in collectivist and hierarchical educational cultures. For example, (36) found that families in several Asian contexts tended to defer responsibility for transition-related decision-making and implementation to schools and professionals, particularly in skill areas requiring formal instruction and certification. This aligns with the present findings, where parents perceived schools as central actors in implementing complex transition skills such as self-advocacy and vocational readiness.

From a systemic perspective, recent international reviews emphasize that institutional structures and policy frameworks can unintentionally limit family engagement in transition implementation. (37), in their review of transition policies, highlighted that even when family involvement is encouraged in policy documents, implementation often remains school-centered due to time constraints, professional role boundaries, and limited parent training opportunities. Similarly, (38) emphasized that effective transition outcomes depend on coordinated professional collaboration, with families supported rather than positioned as primary implementers of specialized transition skills. The present findings suggest that Saudi parents’ perceptions reflect these broader systemic patterns, where schools are viewed as better equipped to carry out transition plans, while families contribute primarily in informal and daily life contexts.

Although no statistically significant differences were found based on gender, educational level, or region, small variations in perception levels were observed. Female parents reported slightly higher perception levels of responsibility compared to male parents. This pattern may be linked to caregiving dynamics within Saudi society, where mothers are more frequently involved in daily educational follow-up and communication with schools. Recent regional research supports this interpretation. (39) reported that Saudi mothers of children with disabilities often assume primary caregiving and coordination roles, which may shape their perceptions of responsibility and engagement in educational processes. While these differences were not statistically significant, they highlight the importance of considering gendered caregiving roles when designing family engagement strategies in transition planning.

The findings also revealed a positive association between perceived school and family responsibilities, indicating that parents who perceived higher levels of school responsibility also tended to perceive higher levels of family responsibility. This relationship suggests that parents do not view school and family roles as competing, but rather as complementary. Such perceptions are consistent with person-centered and collaborative transition frameworks, which emphasize continuity between school-based instruction and family-supported skill reinforcement at home. Strengthening this alignment may be particularly important in contexts where families rely on schools for guidance but remain willing to support implementation when appropriate structures are in place.

### Strengths and limitations

Several limitations should be considered when interpreting these findings. First, the use of self-report measures may introduce response bias, as parents’ perceptions may not fully reflect actual levels of implementation. Second, although the sample included participants from two major regions, the findings may not be generalizable to families in smaller cities or rural areas where access to transition services may differ. Third, participation was voluntary, which may have resulted in the overrepresentation of parents who are more engaged or informed about transition planning. Future studies could address these limitations by incorporating mixed methods, including interviews or observations, and by expanding sampling to include additional regions.

### Implications for practice

Based on the findings, several actionable recommendations can be proposed. At the policy level, the Ministry of Education could develop structured transition training modules targeting both educators and parents, with clear guidance on shared responsibilities across school and home contexts. Introducing parent advocate roles within Individualized Transition Plans may also support more active and informed family participation. Additionally, professional development initiatives for special education teachers could emphasize strategies for engaging families as partners in implementation, particularly in areas such as self-advocacy and vocational skill development. These steps may help reduce the perceived imbalance in responsibility and promote more coordinated transition practices for students with autism in Saudi Arabia.

## Conclusion

This study aimed to examine Saudi parents’ perceptions of responsibility for implementing transition plans for students with autism and to clarify how these responsibilities are distributed between school and family contexts. The findings contribute to the growing body of transition research by providing empirical evidence from a Middle Eastern context, where family–school relationships are shaped by cultural norms, centralized educational systems, and evolving special education policies [34].

The results indicate that parents predominantly perceive schools as the primary agents responsible for implementing transition plans, particularly in areas such as self-advocacy, vocational preparation, and academic skill development, while families are viewed as playing a more substantial role in supporting daily living and independent functioning [34,35]. Rather than reflecting limited parental commitment, this pattern suggests a shared-responsibility model in which roles are differentiated according to perceived expertise and institutional authority. From a theoretical perspective, these findings provide empirical support for shared-responsibility and ecological frameworks of transition planning, particularly within collectivist cultures where formal institutions are often entrusted with structured educational responsibilities.

By situating parental perceptions within the Saudi context, this study extends global transition research that has largely focused on Western settings. It adds a Middle Eastern perspective to the literature, highlighting how cultural expectations, professional authority, and systemic structures influence parental engagement in transition implementation. The consistency of perceptions across gender, educational level, and region further suggests that these views are shaped by broader systemic factors rather than individual characteristics.

At the policy level, the findings underscore the need for clearer operationalization of family roles within transition planning frameworks. While national efforts have expanded special education services, the results point to the importance of nationwide training initiatives that explicitly target both educators and parents. Developing Ministry-level transition training modules, embedding parent advocate roles within Individualized Transition Plans, and strengthening school–family communication mechanisms may help promote more balanced and collaborative implementation practices [36].

From a research perspective, the study highlights the value of comparative investigations across Gulf and Middle Eastern countries to better understand how cultural and policy contexts shape transition practices. Future research could build on these findings by employing mixed-method approaches, examining longitudinal outcomes, and exploring professional perspectives alongside parental views.

In conclusion, this study advances understanding of transition planning by demonstrating how shared responsibility is perceived and enacted within a Saudi context. Aligning transition services with both policy objectives and family realities is essential for improving post-school outcomes for students with autism and for strengthening inclusive transition systems at both national and regional levels [37].

## Supporting information

S1 Table Survey items measuring parental perceptions of responsibility for transition planning. (DOCX)
