# Supplementary material for: Perceptions of Saudi parents of students with autism toward the responsibilities of transition plan members in implementing transition plans
Source: PLoS One. 2026 Mar 20;21(3):e0345501. doi: 10.1371/journal.pone.0345501 (PMC13004325; doi:10.1371/journal.pone.0345501)
Supplement: S1 Table — (DOCX) [file pone.0345501.s001.docx]

### **Supplementary Table S1**

*Survey Items Measuring Parental Perceptions of Responsibility for Transition Planning*

| **1.School Responsibility Items** |
| --- |

| **Item No.** | **Survey Item** |
| --- | --- |
| S1 | The school is responsible for supporting my child’s self-advocacy skills. |
| S2 | The school is responsible for developing my child’s study skills. |
| S3 | The school is responsible for preparing my child for vocational skills. |
| S4 | The school is responsible for supporting my child’s problem-solving skills. |
| S5 | The school is responsible for helping my child set and achieve goals. |
| S6 | The school is responsible for improving my child’s time management skills. |
| S7 | The school is responsible for supporting my child’s decision-making skills. |
| S8 | The school is responsible for developing my child’s social skills. |
| S9 | The school is responsible for developing my child’s self-awareness. |
| S10 | The school is responsible for supporting my child’s independent living skills. |
| S11 | The school is responsible for supporting my child’s interpersonal skills. |
| S12 | The school is responsible for helping my child manage stress. |
| S13 | The school is responsible for supporting my child’s daily living skills. |

| **2.Family Responsibility Items** |
| --- |

| **Item No.** | **Survey Item** |
| --- | --- |
| F1 | My family is responsible for supporting my child’s self-advocacy skills. |
| F2 | My family is responsible for supporting my child’s study skills. |
| F3 | My family is responsible for supporting my child’s vocational skills. |
| F4 | My family is responsible for supporting my child’s problem-solving skills. |
| F5 | My family is responsible for helping my child set and achieve goals. |
| F6 | My family is responsible for supporting my child’s time management skills. |
| F7 | My family is responsible for supporting my child’s decision-making skills. |
| F8 | My family is responsible for supporting my child’s social skills. |
| F9 | My family is responsible for supporting my child’s self-awareness. |
| F10 | My family is responsible for supporting my child’s independent living skills. |
| F11 | My family is responsible for supporting my child’s interpersonal skills. |
| F12 | My family is responsible for helping my child manage stress. |
| F13 | My family is responsible for supporting my child’s daily living skills. |

*Note.* Items were rated on a 5-point Likert scale ranging from 1 (very low responsibility) to 5 (very high responsibility.
